# Mounting Behaviour Recognition for Pigs Based on Deep Learning

**DOI:** 10.3390/s19224924

**Published:** 2019-11-12

**Authors:** Dan Li, Yifei Chen, Kaifeng Zhang, Zhenbo Li

**Affiliations:** College of Information and Electrical Engineering, China Agricultural University, Beijing 100083, China; oliviald@126.com (D.L.); zkaifeng77@126.com (K.Z.); lizb@cau.edu.cn (Z.L.)

**Keywords:** pig, mounting behaviour, deep learning, Mask R-CNN, kernel-extreme learning machine

## Abstract

For both pigs in commercial farms and biological experimental pigs at breeding bases, mounting behaviour is likely to cause damage such as epidermal wounds, lameness and fractures, and will no doubt reduce animal welfare. The purpose of this paper is to develop an efficient learning algorithm that is able to detect the mounting behaviour of pigs based on the data characteristics of visible light images. Four minipigs were selected as experimental subjects and were monitored for a week by a camera that overlooked the pen. The acquired videos were analysed and the frames containing mounting behaviour were intercepted as positive samples of the dataset, and the images with inter-pig adhesion and separated pigs were taken as negative samples. Pig segmentation network based on Mask Region-Convolutional Neural Networks (Mask R-CNN) was applied to extract individual pigs in the frames. The region of interest (RoI) parameters and mask coordinates of each pig, from which eigenvectors were extracted, could be obtained. Subsequently, the eigenvectors were classified with a kernel extreme learning machine (KELM) to determine whether mounting behaviour has occurred. The pig segmentation presented considerable accuracy and mean pixel accuracy (MPA) with 94.92% and 0.8383 respectively. The presented method showed high accuracy, sensitivity, specificity and Matthews correlation coefficient with 91.47%, 95.2%, 88.34% and 0.8324 respectively. This method can be an efficient way of solving the problem of segmentation difficulty caused by partial occlusion and adhesion of pig bodies, even if the pig body colour was similar to the background, in recognition of mounting behaviour.

## 1. Introduction

Intensive interaction among pigs is likely to bring negative effects to pig health as well reduce animal welfare. Mounting behaviour, which occurs to both male and female pigs during their entire lifetime—especially in oestrus [1,2]—is generally manifested as a pig places two front hoofs on the body or head of another pig which stays lying or dodges quickly. Most bruises, lameness and leg fractures are due to mounting behaviour [3] and those injuries will lead to serious economic losses in livestock farming. Therefore, timely detection and intervention of mounting behaviour will be able to increase animal welfare and further ensure pig health.

Traditional monitoring methods of animal behaviour rely mainly on human eye observation which consumes a lot of labour and involves subjective errors. With the development of image and video processing technology, automated video recognition techniques are increasingly applied to pig breeding enterprises. Optical flow vector [4] and fitted ellipse features in consecutive frames [5] were applied to monitoring of the locomotion of pigs by using a charge-coupled device (CCD) camera. For detecting lameness behaviour, joint angle waveform [6] and star skeleton model [7] were utilized from videos taken from the side of the pigs. In order to track the pigs, Kalman filter [8] and modified particle filtering [9] were investigated and had good robustness. Zhu [10] has proposed an automatic detection method for pigs’ respiratory behaviour based on the freeman code algorithm. By monitoring pigs’ appearance in their customary living area, several kinds of behaviours including drinking, feeding and excretory behaviour [11,12] were detected with high overall sensitivity and accuracy. Automatic video processing methods based on fitted ellipse and dense trajectories features [13] can even be utilized for monitoring lying behaviour. A 3D technique has developed rapidly in recent years and more in depth dimension information has been tapped for behaviour detection. Time of flight by Kinect2.0 is used to capture 2D depth images and 3D point cloud images. While Jonguk [14] had taken advantage of 2D depth images in presenting an automatic recognition algorithm for aggressive behaviour detection and Mateusz [15] extracted the cloud point information of pigs from 3D point cloud images and tracked it with ellipsoidal fitting.

Currently, deep learning has received wide attention due to its outstanding performance in computer vision. Sow position was detected from depth images and five classes of sow postures were classified with high overall accuracy using Faster Region-Convolutional Neural Networks (Faster R-CNN) [16,17]. In another survey, images of sows were segmented accurately by a Fully Convolutional Networks (FCN) [18] applied for detecting nursing behaviour. Deep learning has been used for locating pigs in feeding and drinking area and the occupation rate was implemented to measure the feeding and drinking behaviour of pigs using Faster R-CNN and GoogleNet [19,20]. Faster R-CNN was also adopted by [21] while solving the problem of the loss of pig tracking during visual tracking with good robustness and adaptability.

Considering the problems of epidermal injury and fracture caused by mounting behaviour, it is vital to observe the event in time in order to separate the pigs as soon as possible. Computer vision is capable of providing an apposite behaviour recognition method as a solution which is automatic, non-contact, low-cost, high profit and stress-free. An automatic recognition algorithm was implemented by fitting pigs to ellipses and estimating the major and minor axis of each ellipse in order to monitor mounting behaviour [22]. However, the author of this article also expressed that colour-based segmentation has its own weakness. Considering that it is difficult using colour-based segmentation to distinguish a pig’s body from the background and it is also not a simple task to segment the adhesive part among pigs, we applied the deep learning algorithm for pig segmentation. In this research, Mask R-CNN, a deep convolutional network optimized from Faster R-CNN with a branch for predicting an object mask in parallel with the existing branch for bounding box recognition [23], was proposed to segment pigs. Eigenvectors, such as mask perimeter, the half-length mask area divided by the midpoint of long side of the bounding-box and the distance between the centre points of the rectangle and so forth, were extracted for classification to recognize mounting behaviour. The algorithm was applied to male experimental miniature pigs in oestrus and the performance was considerable.

## 2. Material and Methods

### 2.1. Experimental Environment and Animal

**Figure 1:**
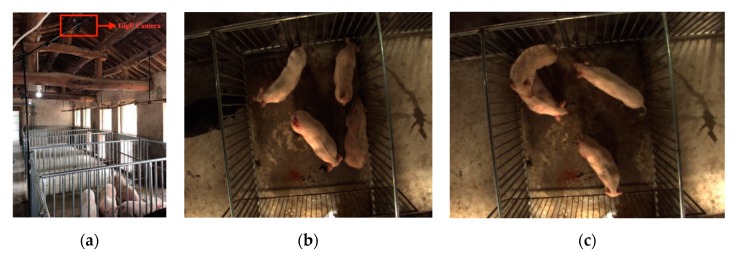
(**a**) Scene of GigE Camera in the pen where the data acquired. (**b**) A sample frame of non-mounting behaviour. (**c**) A sample frame of mounting behaviour.

The experiment was conducted at the China Experimental Miniature Pig Base in Zhuozhou. Four male pigs in oestrus were placed in a pen which was 2 m × 2.2 m in size. The pigs were three months old and about 25 kg in live weight. All lights were switched on during the experiment time, which lasted a week from June 11, 2018 to June 18, 2018. A GigE camera (Allied Vision Technologies, Manta G-282C, Nürnberg, Germany) was mounted on the elevating bracket at about 2.8 m high, pointing downward to get a top view of the pen (Figure 1a). The video acquisition software developed by AVT was selected in order to get video images at the size of 1936 × 1458. Taking into account the image quality and slow moving speed of pigs, the acquisition speed was set at 2 frames per second. All the frames were stored in a mobile hard disk (Western Digital 2Tb ultra, California, United States). Sample frames from the video sequence are shown in Figure 1b.

The pigs used in this trial were four minipigs pigs. This variety is small in size and white in appearance. It can be widely used in the fields of teratogenicity testing, drug metabolism, organ transplantation, skin transplantation test, and so forth. This type of pig has great medical and commercial value, therefore it is vital to detect harmful behaviour like mounting behaviour among pigs in time so that they can be isolated as soon as possible for the avoidance of epidermal damage.

### 2.2. Algorithm Overview

After collecting frames from video sequences, images were pre-processed and put into the network. The pigs’ images were segmented by Mask R-CNN, which performs better than Fully Convolutional Instance-aware Semantic Segmentation (FCIS) [24] on detecting overlapping instances.

**Figure 2:**
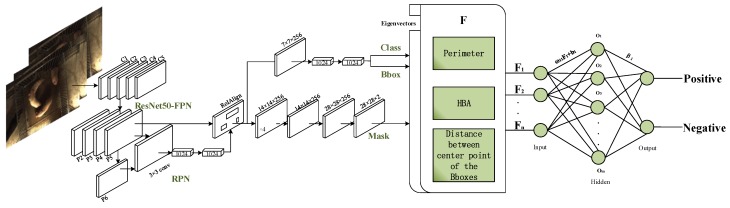
Mounting behaviour detection algorithm overview.

The multi-dimensional eigenvectors were extracted from the parameters of bounding-boxes and the mask files generated from instance segmentation by the pig segmentation network based on Mask R-CNN. Eigenvectors were extracted from the segmentation results and then classified by extreme learning machine. According to the classification result, it is determined whether or not mounting behaviour has occurred. The procedure of this algorithm is shown in Figure 2.

## 3. Algorithm for Mounting Behaviour Detection

### 3.1. Image Pre-Processing and Labelling

After all the video files were observed, 1500 frames were chosen for the mounting behaviour detection experiment. For the purpose of making the sample versatile and contain pigs’ daily behavioural posture, the chosen frames contained a top-down view of feeding, walking, standing, lying, excreting, and mounting behaviours states of pigs in different illumination and cleanliness conditions. The dataset was split randomly into five folds, one fold reserved as test set and the other four reserved as a training set.

**Figure 3:**
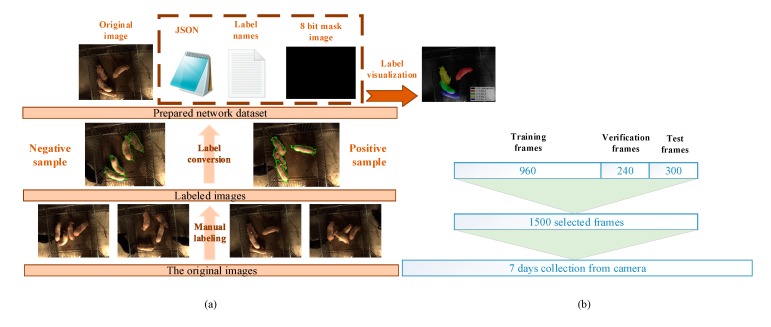
(**a**) Dataset production process. (**b**) Dataset division.

All the selected images were first determined by breeding experts to be divided into positive and negative samples based on whether mounting behaviour had occurred. According to the discriminant result, the frames containing mounting behaviour were taken as positive samples, the rest as negative samples. The research team used polygons to approximately mark the outlines of all the pigs in the images by Labelme toolbox. The JSON files were generated after the annotation. The network ground truth of input label names and masks was converted from JSON files using Labelme as well. The dataset processing procedure is shown in Figure 3a. Dataset allocation is shown in Figure 3b. Considering the sequence frame size obtained from the camera was too large for the network, it was converted to 640 × 480 in pixels.

### 3.2. Pig Segmentation by Mask R-CNN

#### 3.2.1. Related Work

Mask R-CNN is a deep network for instance segmentation proposed by Kaiming He [23]. It is an extension of Faster R-CNN [25]. The prototype of the network dates back to the Region-based CNN (R-CNN) at the 2014 IEEE Conference on Computer Vision and Pattern Recognition (CVPR) [26]. The R-CNN consists of selective search part which proposed 2000 region of interests, CNN and Support Vector Machine (SVM) which combines labels and bounding-box for object classification. Considering that the feature extraction for each candidate region is time consuming and cumbersome, Fast R-CNN [27], which uses a convolutional network to extract features, a RoIPool to extract each RoI corresponding feature in the feature map and an fully connected layer (FC) to classify, were created. However, Faster R-CNN applies a Region Proposal Network (RPN) to optimize the most time-consuming selective search portion, making the entire network a complete end to end network which makes Faster R-CNN flexible and robust. In this paper, we use Mask R-CNN to extract the bounding-boxes and masks of pigs.

#### 3.2.2. The Architecture of Pig Detector

**Figure 4:**
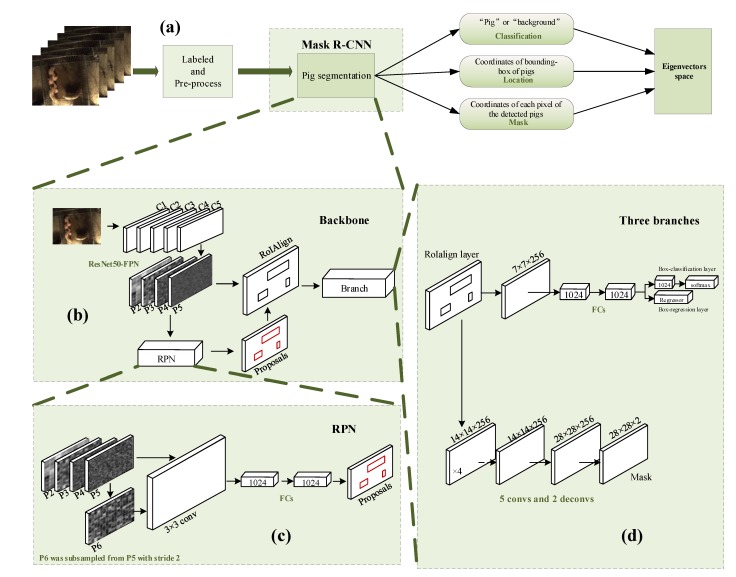
The architecture of pig segmentation network: (**a**) Overflow of the pig segmentation network procedure. (**b**) Close-up of the backbone. (**c**) Close-up of the Reign Proposal Network. (**d**) Close-up of the three branches.

The architecture for the pig segmentation network based on Mask R-CNN is displayed in Figure 4. The entire pig network consists of three parts—(1) a backbone which was the combination of residual neural network and feature pyramid networks with 50 layers (ResNet50-FPN) [28] for feature extraction; (2) an RPN for RoIs proposal; (3) three branches for bbox regression, mask regression and category division.

The pig segmentation network selected ResNet50-FPN as the backbone network. As shown in Figure 4b, the backbone network retained five stages of ResNet50 and generated four-level feature maps as P2~P5. The feature maps were used in RPN for the RoIs proposal. RoIAlign was used for feature extraction. The RPN procedure was shown in Figure 4c, P6 was subsampled from P5 with stride 2. P2~P6 were used for RoIs proposal. The RPN initially determined whether the anchor belongs to the foreground or background and performed the first coordinate correction. In the three branches phrase showed in Figure 4d, in classification and bounding-box regression branches, the output category was firstly set to 2 (pig and background). The full connected layers performed a second parameter correction on the positive bounding-boxes. For obtaining the mask of pigs, the RoIPool was replaced with RoIAlign according to Mask R-CNN. RoIAlign cancels the rounding operation in the process of determining the coordinates in the feature map according to the proposals and the RoIs coordinates which were fixed size (7 × 7) according to the feature map like RoIPool did. Moreover, RoIAlign used bilinear interpolation to find the features corresponding to each block. After being processed by the RoIAlign layer, the mask branch started to use deconvolution to improve the resolution and reduce the channel. Finally, the 28 × 28 × 2 (pig and background) mask was regressed.

#### 3.2.3. The Training and Testing Phase of Pig Segmentation Network

**Figure 5:**
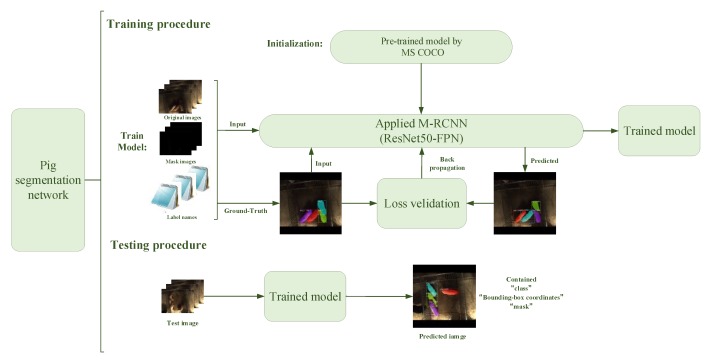
The architecture of the pig segmentation network.

The training procedure of the pig segmentation network is shown in Figure 5.

For solving the problem of difficult training of a small-scale training dataset, the transfer learning method was applied to train the pig segmentation network. The experiment process was as shown.

**Step** **1:** The training dataset containing 1200 samples was randomly split into 5 subsets. One to four folds were reserved as a train set and the remaining was reserved as the validation set. The train set was put into the applied Mask R-CNN.
**Step** **2:** Load the pre-trained model using the Microsoft Common Objects in Context (MS COCO) dataset.
**Step** **3:** Modify the configuration parameters and the number of categories.
**Step** **4:** Start training and observe the change of the validation dataset loss curve.
**Step** **5:** Reset the parameters such as learning rate, weight decay and the anchor scale of RPN, et al.
**Step** **6:** Evaluate pig segmentation network using the validation dataset.
**Step** **7:** Repeat Step 5~Step 6, until the desired accuracy was achieved.
**Step** **8:** Update train set to 2–5 folds and validation set to the first fold. Repeat Step 2~Step 7 five times until the fivefold cross validation were finished and five models were obtained.
**Step** **9:** Using the test set to test five models respectively, the average accuracy and the average Mean Pixel Accuracy (MPA) values were obtained as evaluation metrics of this network.

A file contained each point coordinate of masks and another file contained each bounding-box’s parameter would be generated.

### 3.3. Mounting Behaviour Detection by Kernel-Extreme Learning Machine

#### 3.3.1. Eigenvectors Extraction

After pig segmentation by the Mask R-CNN based network, the mask and bounding-box coordinates could be extracted for eigenvectors acquisition. We extracted the perimeter and the half-body area (HBA) of each pig in the mask as well as the distance between the centre point of every bounding-box in the image as an eigenvector.

**Figure 6:**
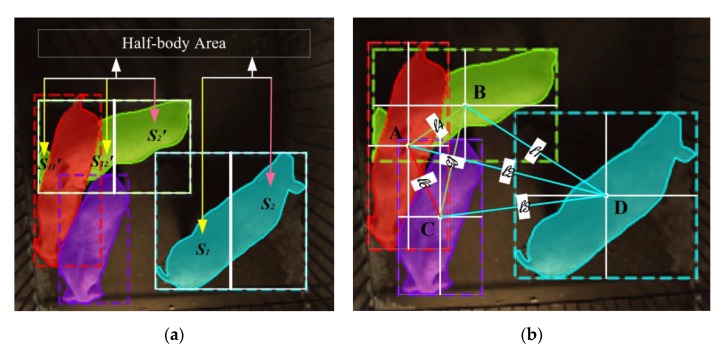
Feature extraction schematic diagram. (**a**) Half-body area schematic: When the pig’s mask is not cut off, half-body area (HBA) is $S_{1}$ and $S_{2}$; Conversely, ${S_{11}}^{\prime }+{S_{12}}^{\prime }$ is the HBA when mounting behaviour occurs. (**b**) Center point spacing schematic: Four pig bounding-box centre points A, B, C, and D and the distance between them was defined as $l_{1}$, $l_{2}$, $l_{3}$, $l_{4}$, $l_{5}$, and $l_{6}$.

(a) HBA: The long side of the bounding-box was found and the area of the mask was divided into two parts $S_{1},\;S_{2}$ with the two long-side midpoint lines as shown in Figure 6a. When the pig’s mask became two parts due to the occurrence of mounting behaviour, the pig’s HBA was defined as $S_{11}^{\prime }+S_{12}^{\prime }$.
(b) The distance between centre point of bounding-boxes (Bboxes) A, B, C, and D in Figure 6b are the centre points of the bounding-boxes of four pigs, respectively. The distance between four points was defined as $l_{1}$, $l_{2}$, …, $l_{6}$. The centre point spacing of the bounding-box frame of each pig in each image was extracted as the eigenvectors.

#### 3.3.2. Classification by Kernel-Extreme Learning Machine

In this article, we used the kernel-extreme learning machine (KELM) to classify the eigenvectors extracted by the Mask R-CNN based pig segmentation network. Kernel-extreme learning machine [29,30] is a kind of machine learning algorithm based on a feedforward neural network. The hidden layer node parameters can be random or artificially given and do not need to be adjusted. It has the characteristic advantages of a short training time and a high generalization ability.

The mounting behaviour detection could be summed up as a two-category problem. In order to establish the classification model of kernel-extreme learning machine, N n-dimensional eigenvectors were recorded as sample $F=\left\{F_{1},F_{2},\ldots ,F_{n,}\right\}$, and the classification result is $T_{1}$ (positive) and $T_{2}$ (negative) represented whether or not the mounting behaviour had occurred.

The mounting behaviour classifier network of the pigs is shown in Figure 6. $\omega_{ij}$ is expressed as the weight between the input neuron i and the hidden layer neuron j, and $b_{j}$ is hidden layer neuron offset. The hidden layer activation function is g(·) and $O_{1}$, $O_{2}$, …, $O_{m}$ are hidden layer nodes.

The output T in the model can be expressed as: T=Hβ. H is the hidden layer output matrix and β is the weight between hidden layer and output layer.

(1) $$H\left(\omega_{1},\ldots ,\omega_{m},b_{1},\ldots ,b_{m},F_{1},\ldots ,F_{m}\right)={\left[\begin{array}{ccc} g\left(\omega_{11}F_{1}+b_{1}\right) & \cdots & g\left(\omega_{1m}F_{1}+b_{m}\right)\\ \vdots & \cdots & \vdots \\ g\left(\omega_{1N}F_{N}+b_{1}\right) & \cdots & g\left(\omega_{Nm}F_{N}+b_{m}\right) \end{array}\right]}_{N\times m}$$

(2) $$\beta ={\left[\begin{array}{c} \beta_{1}^{T}\\ \vdots \\ \beta_{m}^{T} \end{array}\right]}_{m\times 2}$$

(3) $$T={\left[\begin{array}{c} T_{1}^{T}\\ \vdots \\ T_{N}^{T} \end{array}\right]}_{N\times 2}$$

(4) $$T_{i}=\sum_{j=1}^{m}\beta_{j}g\left(\omega_{ij}F_{i}+b_{j}\right),\;i=1,2,\ldots ,N$$

Given an arbitrary small error ε (ε>0) and an arbitrary interval infinitely differentiable activation function g(·), there is always an ELM with m hidden layers, arbitrarily assigned $\omega_{ij}\in {\mathbb{R}}^{n}$ and $b_{j}\in \mathbb{R}$, there is:

(5) ∥Hβ−T∥<ε

When hidden layer activation function g(·) can be guided, the ELM network does not need to change all the parameters and solves the Equation (5):

(6) $$\tilde{\beta }=H^{+}T,$$

where $H^{+}$ is the generalized inverse matrix of the hidden layer output matrix T; $\tilde{\beta }$ is the output layer weight.

A kernel matrix for ELM could be defined as follows:

(7) $$\Omega_{ELM}=HH^{T}:\Omega_{ELMi,j}=h\left(x_{i}\right)\cdot h\left(x_{j}\right)=K\left(x_{i},x_{j}\right).$$

Then, the output function of ELM classifier can be written compactly as:

(8) $$f\left(x\right)=h\left(x\right)H^{T}{\left(\frac{1}{C}+HH^{T}\right)}^{-1}T=\left[\begin{array}{c} K\left(x,x_{1}\right)\\ \vdots \\ K\left(x,x_{N}\right) \end{array}\right]{\left(\frac{1}{C}+\Omega_{ELM}\right)}^{-1}T$$

Therefore, when training the mounting behaviour classifier, the feature mapping h(x) need not be known to users; instead its corresponding kernel K (**u**, **v**). The test results will be obtained by applying the initial hidden layer weights and the output layer weights obtained by the training.

#### 3.3.3. The Training and Testing Phase of Extreme Learning Machine

K-fold cross validation has been widely used to evaluate the performance of machine learning classifiers. In order to seek the hyperparameters, we tuned the parameter based on fivefold cross validation and grid search. That is to say, the training dataset was randomly split into five subsets, and one of those sets was reserved as a test set and the others are reserved as a train set. This process would be repeated five times until getting five optimal models.

After obtaining five models, the models would be tested by the preserved test set. The average of the result would be used to evaluate the performance of the mounting behaviour recognition classifier.

### 3.4. Performance Evaluation of the Mounting Behaviour Recognition Method

The performance of the pig segmentation network is evaluated by the commonly used metric, Mean Pixel Accuracy (MPA). It is a standard measurement in the evaluation of image segmentation and it is derived from the currently segmented pixel:

(9) $$Mean\;Pixel\;Accuracy\left(MPA\right)=\frac{1}{k+1}\sum_{i=0}^{k}\frac{P_{ii}}{\sum_{j=0}^{k}P_{ij}}$$

where k is the total number of categories except the background, $P_{ij}$ is the total number of the pixel whose real class is i and predicted as j, and $P_{ii}$ is the total number of pixels whose real pixel class is i and collectedly predicted.

To evaluate the performance of the prediction model of mounting behaviour recognition, four metrics [31,32,33] were utilized—Specificity(SP), sensitivity(SN), accuracy(ACC), and the Matthews correlation coefficient (MCC), which are defined as follows:

(10) $$\begin{array}{c} Specificity\left(SP\right)=\frac{TN}{TN+FP}\\ Sensitivity\left(SN\right)=\frac{TP}{TP+FN}\\ Accuracy\left(ACC\right)=\frac{TP+TN}{TP+FP+TN+FN}\\ Matthews\;correlation\;coefficient\left(MCC\right)=\frac{TP\times TN-FP\times FN}{\sqrt{\left(TP+FN\right)\left(TP+FP\right)\left(TN+FP\right)\left(TN+FN\right)}} \end{array}$$

where *TP* (true positive) represents the number of positive samples predicted to be positive; *TN* (true negative) represents the number of negative samples predicted to be negative; *FP* (false positive) represents the number of negative samples predicted to be positive; *FN* (false negative) represents the number of positive samples predicted to be negative.

## 4. Result

### 4.1. Experiment and Evaluation of Pig Segmentation

The experiment was done by using Tensorflow and Keras deep learning frameworks in python, with NVIDIA TITAN RTX (NVIDIA, Santa Clara, CA, USA) for acceleration. The datasets are strictly non-intersecting with each other. The network was initialized with an MS COCO pre-trained model. Then, all the layers were fine-tuned according to the new pig dataset. The RPN proposal number was 2000 and the positive ratio was 0.33. The learning rate was 0.0005. The weight decay was 0.00005 and the momentum was 0.9.

**Figure 7:**
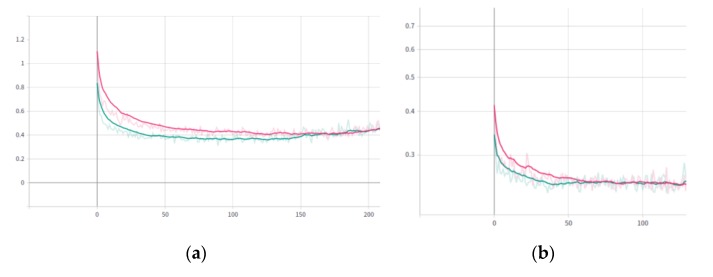
Validation dataset loss curve during training. (**a**) Validation dataset loss curve (including classification, bounding-box regression and mask loss): the abscissa axis value represented epoch, and the ordinate axis value represented the loss value. (**b**) Mask loss curve of the validation dataset: the abscissa axis value represented epoch, and the ordinate axis value represented the loss value.

In order to compare the pig segmentation network effects, we chose ResNet50-FPN and ResNet101-FPN as backbone networks. The lumped loss changes and the mask loss changes during the training are shown in Figure 7. Among them, red curve indicated that the backbone network was ResNet50-FPN and the green curve represented ResNet101-FPN.

**Figure 8:**
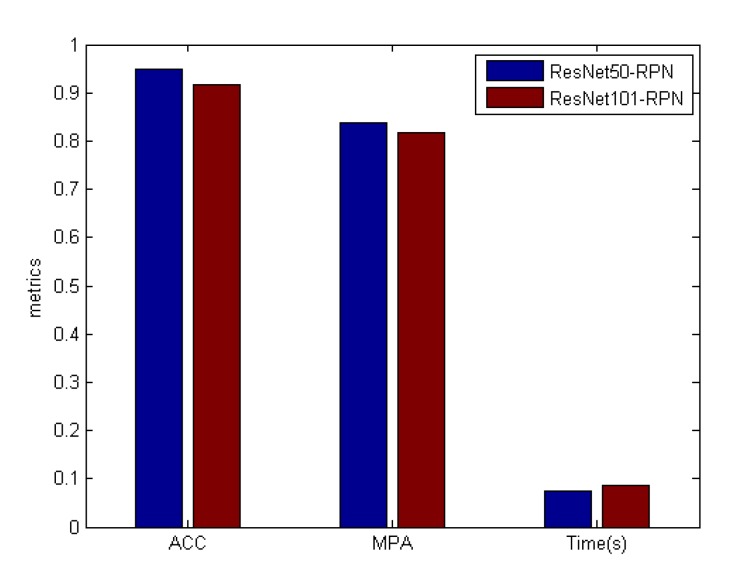
Performance of the ResNet50-FPN and the ResNet101-FPN based pig segmentation network. The accuracy represents the ratio of the images correctly recognized by the network (four pigs). MPA is the average value of each image. Time represents the average detection time of each image.

As can be seen from Figure 7, ResNet101 took fewer epochs than ResNet50 before convergence but took more time (15 min 24 s). ResNet50 took 18 s per epoch which was 6s less than ResNet101. It can be concluded that ResNet50-FPN converge faster than ResNet101-FPN as a backbone in the pig segmentation network. The pig segmentation network was tested with the test dataset. The result is shown in Figure 8.

**Figure 9:**
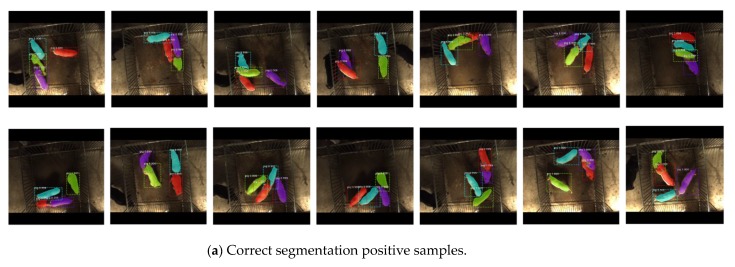

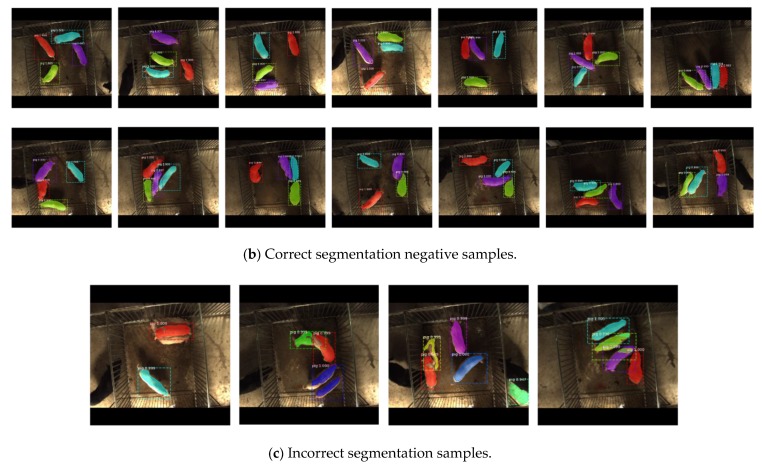
Samples of pig segmentation result: (**a**) pig segmentation while mounting behaviour occurred; (**b**) pig segmentation without mounting behaviour; (**c**) incorrect segmentation.

The test samples were analysed. Among 300 samples, there were 1200 pigs including 305 pigs suspected of mounting behaviour, 113 pigs involved in interaction adhesion, 782 separated from each other. It was shown in Table 1 that in terms of pig detection, the pig recognition accuracy of ResNet50-FPN was 94.92% which was 3.26% higher than that of ResNet101-FPN network in five models trained by different train sets. In terms of pig segmentation, the MPA of ResNet50-FPN was 0.8383 which was 0.022 higher than that of ResNet101. In terms of running time, it took 0.012 more seconds for each image in the process of detection and segmentation in ResNet101. Some segmentation results based on Mask R-CNN with ResNet50-FPN backbone samples are shown in Figure 9.

Considering the above data, it could be inferred that the pig segmentation network was effective in pig detection and segmentation while partial occlusion and adhesion of the pig body occurred even if the pig bodies’ colour was similar to the background. The reason the MPA was not extremely high is due to the irregular phenotype of the mounted pig. In fact, both the mounted pig and other pigs were labelled ‘pig,’ which made the mask branch more difficult to converge. Although ResNet101-FPN has a stronger ability for complex fitting, it was too deep for one category and it easily caused the over fitting. Therefore, we chose ResNet50-FPN as the backbone of the pig segmentation network.

Considering the above data, it could be inferred that the pig segmentation network was effective in pig detection and segmentation while partial occlusion and adhesion of the pig body occurred even if the pig bodies’ colour was similar to the background. The reason the MPA was not extremely high is due to the irregular phenotype of the mounted pig. In fact, both the mounted pig and other pigs were labelled ‘pig,’ which made the mask branch more difficult to converge. Although ResNet101-FPN has a stronger ability for complex fitting, it was too deep for one category and it easily caused the over fitting. Therefore, we chose ResNet50-FPN as the backbone of the pig segmentation network.

**Figure 10:**
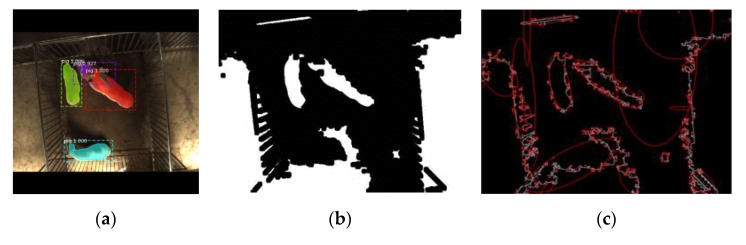
Comparison between Mask R-CNN and traditional segmentation method: (**a**) Image segmentation by pig segmentation network. (**b**) Image segmentation by threshold (Otsu). (**c**) ellipse fitting.

Actually, when dealing with some images with overlapping pigs, the recognition rate while using the pig segmentation network was indeed better than that of the traditional algorithm. Figure 10 showed pigs extracted by the pig segmentation network and traditional threshold segmentation. Due to the illumination, the traditional method failed to recognize the pig in the bottom part of the image and identified some part of the bright ground as part of the pig’s body. Also, the two overlapping pigs were even regarded as one. It could be inferred that the pig segmentation network was superior to traditional methods in the recognition of overlapping pigs in the case where the background was very similar to the colour of the pig in images.

After analysis, we hold the opinion that the cause of the error was related to the stage of mounting behaviour. Considering that the images were taken from video, the samples could be extracted from any stage of mounting behaviour. During the early or middle stage of mounting, the pigs involved in the mounting behaviour had a large possibility of being recognized. However, if the image was taken during the late stage of mounting behaviour, it would be difficult to segment the outlines of the pigs because the overlapping area was extremely large.

### 4.2. Experiment and Evaluation of Mounting Behaviour Classifier

In order to keep the balance of positive and negative samples in the training set, we randomly reduced the negative samples in the original training set by 146, and 1054 eigenvectors were extracted for the training process of KELM. The KELM was done in Matlab (R2014a) (MathWorks, Natick, MA, USA) with an Intel Core i9-9900k CPU (Intel Corporation, Santa Clara, CA, USA). The accuracy of the test dataset classified by four different machine learning classifiers were compared and the results were shown in Table 2.

The commonly used four different machine learning algorithms were explored, including back propagation neural network (BP), Random forest (RF), Extreme learning machine (ELM) and Kernel-Extreme learning machine (KELM). Table 2 represents the performance of the four different classifiers on mounting behaviour recognition. The values were obtained by calculating the average values of the preserved test result. BP, RF, ELM and KELM respectively achieved an average MCC of 0.7917, 0.7962, 0.8158 and 0.8324. In particular, KELM showed a 1.67–4.07% higher MCC score than that of other three classifiers. By comparing with BP, RF and ELM, the accuracy and sensitivity of the KELM was 0.74–1.94% and 2.22–5.46% higher. However, the RF performed better in specificity with 1.19–2.25% compared with the other three classifiers. Hence, we considered the KELM classifier for mounting behaviour recognition of pigs.

It can be seen from the analysis results above that the proposed algorithm is efficient and has considerable accuracy (91.47%), sensitivity (95.2%), specificity (88.34%) and Matthews correlation coefficient (0.8324) in mounting behaviour recognition. It is noted that, because the pig segmentation network could not segment all the images accurately and completely, the dataset used to train KELM was not complete or accurate and some small parts were even missing. However, KELM still showed a good classification effect. Moreover, the proposed algorithm has strong portability. When the application environment changes, it will be capable of getting a considerable result after re-collecting new images for transfer learning.

## 5. Conclusions

In this study, we proposed a new algorithm for mounting behaviour recognition of pigs based on deep learning. The algorithm contained three parts—the pig segmentation network, eigenvectors extraction and KELM.

(a) The pig segmentation network based on Mask R-CNN was applied and evaluated. The results showed that taking ResNet50-FPN as the backbone got better accuracy and Mean Pixel Accuracy which were 94.92% and 0.8383. This pig segmentation model can effectively solve the problem of segmentation difficulty caused by partial occlusion and adhesion of the pig body even if the pig bodies’ colour was similar to the background.
(b) We proposed three features extracted in each image for getting the eigenvector—the perimeter of each pig, the half-body area (HBA) of each pig’s mask and the distance between the centre point of every bounding-box in one image.
(c) The complete algorithm was evaluated by external validation and the experiment result showed that this method was efficient and the performance of the algorithm has considerable accuracy (91.47%), sensitivity (95.2%), specificity (88.34%) and Matthews correlation coefficient (0.8324) in mounting behaviour recognition.

Considering the information limitations of visual images, the deep image can be an alternative for further research into detecting mounting behaviour. The result should be even more impressive.

## Figures and Tables

**Table 1:** Performance of the ResNet50-FPN and ResNet101-FPN based pig segmentation network.

Methods	ACC	MPA	Time (s/pic)
ResNet50-FPN	0.9492	0.8383	0.0746
ResNet101-FPN	0.9166	0.8163	0.0866

**Table 2:** Comparison of the precision rate of machine learning classifiers.

Methods	ACC	MCC	SN	SP
BP neural network	0.8953	0.7917	0.9161	0.8766
Random forest	0.898	0.7962	0.8974	**0.8991**
Extreme learning machine	0.9073	0.8158	0.9298	0.8872
Kernel-ELM	**0.9147**	**0.8324**	**0.9520**	0.8834
